# RAGE is a critical factor of sex-based differences in age-induced kidney damage

**DOI:** 10.3389/fphys.2023.1154551

**Published:** 2023-03-29

**Authors:** Seerat Bajwa, Alexander Luebbe, Ngoc Dong Nhi Vo, Eva-Maria Piskor, Christian Kosan, Gunter Wolf, Ivonne Loeffler

**Affiliations:** ^1^ Department of Internal Medicine III, Jena University Hospital, Jena, Germany; ^2^ Institute of Biochemistry and Biophysics, Center for Molecular Biomedicine (CMB), Friedrich Schiller University, Jena, Germany

**Keywords:** renal damage, aging, inflammation, sex-based difference, AGE (advanced glycation end products), RAGE (receptor for advanced glycation end products)

## Abstract

**Introduction:** Advanced glycation end products (AGEs) are a heterogeneous group of molecules with potential pathophysiological effects on the kidneys. Fibrosis together with the accumulation of AGEs has been investigated for its contribution to age-related decline in renal function. AGEs mediate their effects in large parts through their interactions with the receptor for AGEs (RAGE). RAGE is a transmembrane protein that belongs to the immunoglobulin superfamily and has the ability to interact with multiple pro-inflammatory/pro-oxidative ligands. The role of RAGE in aging kidneys has not been fully characterized, especially for sex-based differences.

**Methods:** Therefore, we analyzed constitutive RAGE knockout (KO) mice in an age- and sex-dependent manner. Paraffin-embedded kidney sections were used for histological analysis and protein expression of fibrosis and damage markers. RNA expression analysis from the kidney cortex was done by qPCR for AGE receptors, kidney damage, and early inflammation/fibrosis factors. FACS analysis was used for immune cell profiling of the kidneys.

**Results:** Histological analysis revealed enhanced infiltration of immune cells (positive for B220) in aged (>70 weeks old) KO mice in both sexes. FACS analysis revealed a similar pattern of enhanced B-1a cells in aged KO mice. There was an age-based increase in pro-fibrotic and pro-inflammatory markers (IL-6, TNF, TGF-β1, and SNAIL1) in KO male mice that presumably contributed to renal fibrosis and renal damage (glomerular and tubular). In fact, in KO mice, there was an age-dependent increase in renal damage (assessed by NGAL and KIM1) that was accompanied by increased fibrosis (assessed by CTGF). This effect was more pronounced in male KO mice than in the female KO mice. In contrast to the KO animals, no significant increase in damage markers was detectable in wild-type animals at the age examined (>70 weeks old). Moreover, there is an age-based increase in AGEs and scavenger receptor MSR-A2 in the kidneys.

**Discussion:** Our data suggest that the loss of the clearance receptor RAGE in male animals further accelerates age-dependent renal damage; this could be in part due to an increase in AGEs load during aging and the absence of protective female hormones. By contrast, in females, *RAGE* expression seems to play only a minor role when compared to tissue pathology.

## 1 Introduction

Aging is a multifactorial process characterized by progressive decline in the physiological function of many organs (Evans, 2000). Age-related renal impairment has become an impending challenge to clinical practice. This process is associated with macroscopic and microscopic histological alterations such as glomerulosclerosis caused by glomerular basement membrane thickening (Sato and Yanagita, 2019; Birch and Gil, 2020) and mesangial expansion because of enhanced accumulation of the extracellular matrix, decrease in cortical tissue, tubular atrophy, and interstitial fibrosis (Zhou et al., 2008; Sethi et al., 2017). One of the hallmarks of an aging kidney is cellular senescence, which is a permanent cell cycle arrest that is characterized by the accumulation of cells with a senescent phenotype. These senescent cells secret a specific senescence-associated secretory phenotype (SASP), which includes pro-inflammatory cytokines, mainly interleukin (IL)-1α, IL-1β, IL-6, and IL-8, several growth factors (e.g., hepatocyte growth factor, transforming growth factor-β, and granulocyte–macrophage colony-stimulating factor), and chemokines, and are responsible for age-associated low-grade inflammation (Sato and Yanagita, 2019; Birch and Gil, 2020). These structural and functional abnormalities associated with age inhibit clearance and tissue regeneration, thus increasing the risk to permanent renal damage (D O'Sullivan et al., 2016). In addition, advanced age increases susceptibility to various renal diseases (Sturmlechner et al., 2017) such as acute kidney injury (AKI) (O’Sullivan et al., 2017; Saran et al., 2018), chronic kidney disease (CKD), and diabetic nephropathy (Martin and Sheaff, 2007). Epidemiologic studies suggest that age-related decline of renal function may be accelerated by several factors, such as inflammation (Fried et al., 2004), increased levels of advanced glycation end products (AGEs) (Vlassara et al., 2009a), and male gender (Baylis, 2009).

AGEs are a heterogeneous, complex group of compounds that are formed by non-enzymatic glycation and oxidation of proteins and lipids (Maillard, 1912). This process known as the Maillard reaction has already been identified more than 100 years ago, in which starting with the soluble Schiff base, an intermediate stable Amadori product, and then an irreversible aggregate (AGEs) are all formed (Dyer et al., 1991; Vistoli et al., 2013). Glycated hemoglobin (HbA1c) is among the first observed endogenous glycation product (Rahbar, 2005). AGEs formation is accelerated under hyperglycemic and/or hypoxic conditions and contributes to the structural changes of macromolecules (Bohlender et al., 2005). Antibodies against AGE-specific epitopes have shown that accumulation of AGEs in human tissues under physiological conditions, with excessive accumulation, are known to progress with chronological aging and become associated with disturbed renal function in the elderly (Dyer et al., 1993), especially in diabetic patients where AGEs concentration is increased threefold (Ahmed et al., 2005).

Several receptors for AGEs have been identified; however, the receptor for advanced glycated end products (RAGE) is the best characterized receptor of AGEs. It is a type I cell surface receptor belonging to the immunoglobulin (Ig) superfamily (Neeper et al., 1992). AGEs bind to RAGE and trigger signaling cascades, which leads to the production of reactive oxygen species (ROS), pro-inflammatory factors, and acute phase proteins (Stern et al., 2002; Busch et al., 2010; Perrone et al., 2020). Several previous studies have shown that deletion of RAGE has a protective effect in the context of diabetic nephropathy, neuropathy, angiogenesis, and arteriosclerosis (Wendt et al., 2003; Yonekura et al., 2003; Shoji et al., 2006; Soro-Paavonen et al., 2008), while its activation leads to podocyte hypertrophy and glomerular inflammation (Liebisch et al., 2014). Other AGE receptors, like AGE-R1, AGE-R2, AGE-R3, and class A macrophage scavenger receptor types I and II (MSR-AI/II), also recognize and bind AGE ligands, but they do not start signal transduction after binding to AGE (Stitt et al., 1997; Bucciarelli et al., 2002); on the other hand, they might have a role in detoxification and clearance of AGEs (Bucciarelli et al., 2002). AGE-R1 is a type 1 single transmembrane protein, which has a small extracellular N-terminal domain and a cytoplasmic C-terminal domain (Yang et al., 1991; Kelleher et al., 1992). It is known that AGE-R1 lowers the intra- and extra-cellular AGEs level and facilitates its clearance through urine (Vlassara et al., 2009b; Uribarri et al., 2011). AGE-R2 is an 80- to 90-kD protein that mediates the intracellular signaling of various receptors, such as the fibroblast growth factor receptors (Goh et al., 1996; Stitt et al., 1997). AGE-R2 contains a tyrosine-phosphorylated region in the plasma membrane of the cell (Li et al., 1996a; Goh et al., 1996). The AGE-R3–binding domain is at the C-terminus, and it binds with high affinity to AGE ligands (Vlassara et al., 1995). MSR-A seems to be involved in AGE uptake and in the endocytic degradation of AGE proteins by macrophages and macrophage-derived cells (Pugliese et al., 2001; Batzdorf et al., 2022). Two class B scavenger receptors—CD36 and class B type I—also bind AGE ligands. CD36 plays a vital role in the induction of oxidative stress in cells but is not involved in the clearance of AGEs (Horiuchi et al., 2005; Nakajou et al., 2005).

There is a complex relationship between gender and kidney diseases, with men and women having different biological susceptibilities to the disease (Murphy et al., 2016), and this difference has recently been emphasized (Health NIo, 2001). Recent studies have shown that although more women than men have chronic kidney disease (CKD), there are greater chances of men reaching kidney failure sooner than women. For this reason, being male is used as a risk factor to predict a faster time in reaching kidney failure with greater severity (Ricardo et al., 2019; Harris and Zhang, 2020). The reason for these gender differences is not clearly understood. Many animal studies have shown that this sexual disparity is due to sex hormones. Higher testosterone levels and epidermal growth factor receptor (EGFR) may cause loss in kidney function in male mice (Doublier et al., 2011; Hewitson et al., 2016; Harris and Zhang, 2020). By contrast, estrogen is known to have a protective effect on kidney health (Feng et al., 2014), thus highlighting the need for including both sexes in studies to provide a better understating of scientific questions.

The importance of RAGE in the context of aging has been investigated before (Teissier et al., 2019), but sex-based difference in the context of aging has not been characterized yet. This study provides the first attempt of the potential role of RAGE expression in both sexes and its contribution to renal pathology in an age-dependent manner.

## 2 Materials and methods

### 2.1 Animals

*RAGE* ^−/−^ mice (B6;129S5-Ager<tm1Lex>/leg (LEXKO-2071) were obtained from EMMA (Helmholtz Zentrum Munich, German Research for Environmental Health, Munich, Germany). The mice used in this study were first backcrossed to the C57BL/6 background for more than 10 generations and later to the C57BLKS background. Age-matched wild-type mice from the same breeding cohort were used as the control. The mice were kept in a pathogen-free animal house with 12-h light/dark cycle on standard chow and water *ad libitum*. All experiments were performed in accordance with the guidelines of the German Animal Welfare Act (§7). For histopathology and RNA expression (*n* = 3–7 per group), the mice were divided into young (<30 weeks) and old (>70 weeks), and to study sex-based differences, male and female mice were also included. For flow cytometric analysis (*n* = 4) (62–68 weeks), both male and female mice were grouped together.

### 2.2 Histopathological analysis

For histological analysis, the kidneys were cut in half, sagittal, and fixed in 10% neutral buffered formalin with subsequent embedding in paraffin. The paraffin-embedded tissues were sliced at 4 µm thickness, dewaxed, rehydrated, and stained with hematoxylin and eosin (H&E) and Masson’s trichrome stains using the standard protocol. For glomerular and tubular damage, H&E sections were assessed with a score of 0–4 (0 = normal glomerular structure, no interstitial and tubular damage; 1 = 10%, 2 = 25%, 3 = 50%, and 4 = >60%). For renal fibrosis, Masson’s staining was visually assessed for collagen blue staining in the glomeruli and in the interstitium and quantified with a score of 0–5 (0 = no blue staining; 1 = <10%, 2 = 10–20%, 3 = 20–40%, 4 = 40–60%, and 5 = >60%). At least 10–15 images were captured for each section and quantified for histopathological changes. Images were acquired using AxioVision 4.8 software with an AxioCam HRc camera (Zeiss, Jena, Germany).

### 2.3 Immunofluorescence staining

#### 2.3.1 B220 staining

Sections of 4 µm were deparaffinized, rehydrated, and heat-mediated antigen retrieved with citrate buffer (pH 6). After blocking with 10% BSA (bovine serum albumin, Roth, Karlsruhe, Germany) for 1 h at room temperature, primary antibody [anti-mouse B220 monoclonal antibody (1:200), eBioscience/Invitrogen] incubation was done in a humid chamber overnight at 4°C. The sections were stained with suitable secondary antibodies [anti-mouse IgG, Alexa Fluor 488, Thermo Fisher Scientific (1:500)] for 1 h at room temperature and mounted using DAPI mounting medium (Vector Laboratories, Burlingame, CA, United States). Images were acquired using AxioVision 4.8 software with an AxioCam HRc camera (Zeiss, Jena, Germany).

#### 2.3.2 AGE staining

Sections of 3 µm were deparaffinized, rehydrated, and heat-mediated antigen retrieved with citrate buffer (pH 9). Staining with primary antibodies (rabbit anti-mouse AGE polyclonal antibodies (1:400), Bioss Antibodies, Woburn, MA, United States) and DAPI was done with opal fluorochrome–based staining chemistry AKOYA Opal 4-Color Anti-Rabbit Manual IHC Kit (Phenoptics™/Akoya Biosciences, Marlborough, MA, United States). The images were taken using the multispectral scanner Vectra Polaris™ (Akoya Biosciences) at the Institute of Forensic Medicine, Pathology Department, University Hospital Jena, visualized with the software Phenochart™ 1.1 (Akoya Biosciences, Marlborough, MA, United States), and quantified using a score of 0–5 (0 = no accumulation; 1 = weak accumulation, 2 = mild accumulation, 3 = moderate accumulation, 4 = strong accumulation, and 5 = very strong accumulation).

### 2.4 Immunohistochemistry

#### 2.4.1 NGAL staining

Sections of 4 µm were deparaffinized, rehydrated, and heat-mediated antigen retrieved with citrate buffer (pH 6). The sections were blocked for endogenous peroxidase activity using 0.03% H_2_O_2_ (Roth, Karlsruhe, Germany) for 30 min at room temperature followed by blocking with 10% BSA (Roth, Karlsruhe, Germany) for 1 h at room temperature. Primary antibody (goat-polyclonal, anti–lipocalin-2/NGAL (1:200) R&D system, Minneapolis, United States) incubation was done overnight at 4°C. After incubation with peroxidase-labeled secondary antibodies [rabbit anti-goat IgG, SeraCare Life Sciences, Milford, United States (1:1,000)] for 1 h, ImmPACT NovaRED (peroxidase substrate kit; Vector Laboratories, Burlingame, CA, United States) was used as a chromogen. At least 10–15 images were captured for each section and quantified for NGAL tubular expression visually using a score of 0–5 (0 = <5%; 1 = 10%, 2 = 25%, 3 = 50%, 4 = 75%, and 5 = <75%). Images were acquired using AxioVision 4.8 software with an AxioCam HRc camera (Zeiss, Jena, Germany).

In addition to visual quantification, staining was quantified using an ImageJ Macro in an automated manner. In brief, Zeiss Vision Image (.zvi) format was exported to TIFF format. The gained data were analyzed by using the thresholding function of ImageJ. For each staining, an individual threshold was customized and applied to all images of that staining. Staining was segmented into relevant and irrelevant. The obtained data of staining intensity per relevantly stained area per image were averaged for each mouse.

#### 2.4.2 CTGF staining

For CTGF staining, 3 µm sections were deparaffinized and rehydrated. No heat mediated antigen retrieval was performed. Blocking of endogenous peroxidase was achieved by incubation with 3% H_2_O_2_ (Roth, Karlsruhe, Germany) for 10 min at room temperature. Following the blocking with Roti-Block (Roth, Karlsruhe, Germany), the sections were incubated with primary rabbit polyclonal anti-mouse CTGF antibodies (Abcam, Cambridge, United Kingdom) overnight at 4°C. After incubation with peroxidase-labeled goat anti-rabbit IgG antibody (SeraCare, Milford, MA, United States), diaminobenzidine (DAB) (DAB-peroxidase substrate kit; Vector Laboratories, Burlingame, CA, United States) was used as a chromogen. Whole-slide images were taken using the multispectral scanner Vectra Polaris™ (Akoya Biosciences) by Pathology Department, University Hospital Jena, visualized with software Phenochart 1.1 (Akoya Biosciences, Marlborough, MA, United States) and quantified with a score of 0–5 (0 = no fibrosis; 1 = weak fibrosis, 2 = mild fibrosis, 3 = moderate fibrosis, 4 = strong fibrosis, and 5 = very strong fibrosis).

### 2.5 Analysis of renal TGF-β1 protein expression

Total protein from the cortices of mouse kidneys (*n* = 4 per group) was isolated by homogenization with complete Lysis-M buffer supplemented with protease inhibitors (both from Roche Diagnostics, Mannheim, Germany). After centrifugation, the activated TGF-β1 concentrations in the protein lysates of the renal tissue were quantitatively determined by using the Mouse/Rat/Porcine/Canine TGF-β1 ELISA according to the manufacturer’s instructions (Quantikine, R&D Systems, Wiesbaden, Germany).

### 2.6 Flow cytometry

Single-cell suspensions from kidney tissues were generated at the time of autopsy. The kidneys were dissociated using the Multi Tissue Dissociation Kit 2 (Miltenyi Biotec) and gentleMACS™ Octo Dissociator with Heaters (Miltenyi Biotec) according to the manufacture’s recommendations. After dissociation, cell debris was removed from the kidney suspensions using a debris removal solution (Miltenyi Biotec).

For flow cytometry, 1 × 10^6^ cells from single-cell suspensions were used for antibody staining. Staining was performed at 4°C for 15 min in PEB buffer (PBS with 2 mM EDTA and 0.5% FCS). Following antibodies were used in the staining: CD45R/B220 (RA3-6B2), CD19 (eBio1D3), CD43 (S7) from eBioscience/Invitrogen, CD19 (1D3), CD3 (500A2), NK1.1 (PK136), CD11b (M1/70) from BioLegend, CD5 (REA421) and CD45 (REA737) from Miltenyi Biotec B.V. & Co. KG, FSC and SSC signals were acquired to eliminate dead cells and doublets. The resulting population was defined as living cells. Data were acquired using the BD LSRFortessa™ and BD FACSDiva™ v8.0.1 (BD Biosciences). Analyses were performed using FlowJo™ v10.8 (Becton, Dickinson and Company).

### 2.7 cDNA synthesis and semi-quantitative real-time PCR

Total RNA from the kidney cortex was isolated using the NucleoSpin 8 RNA Kit (Macherey–Nagel, Düren, Germany). Possible DNA contamination was eliminated using the RNase-Free DNase Set (QIAGEN, Hilden, Germany), and 1 µg total RNA was reverse transcribed into cDNA with the Reverse Transcription System (Promega, Madison, WI, United States). Gene expression was assessed by semi-quantitative real-time PCR by LightCycler FastStart DNA Master SYBR Green 1 (Roche Diagnostics, Mannheim, Germany) using a thermocycler (qTOWER^2^, Analytik Jena, Germany). PCRs were carried out with sense and antisense primers at a concentration of 0.25 µM each (TIB MOLBIOL, Berlin, Germany). Temperatures and sequences of all primer pairs are shown in Table 1. Hypoxanthine phosphoribosyltransferase 1 (HPRT1) was used as the housekeeping gene. The relative expression ratio was quantified by the ∆∆CT method, and the transcript levels were normalized to the mean value of the young male wild-type control group.

**TABLE 1:** Gene primers and their respective annealing temperature.

Gene	Sense and antisense primers	Tann
*NGAL*	5′-CAC​CAC​GGA​CTA​CAA​CCA​GTT​CGC-3′	59°C Mishra et al. (2003)
	5′-TCA​GTT​GTC​AAT​GCA​TTG​GTC​GGT​G-3′	
*KIM1*	5′-ATG​AAT​CAG​ATT​CAA​GTC​TTC-3′	58°C Bai et al. (2016)
	5′-TCT​GGT​TTG​TGA​GTC​CAT​GTG-3′	
*IL-6*	5′-ACA​AGT​CCG​GAG​AGG​AGA​C-3′	58°C Hammerschmidt et al. (2009)
	5′-CAG​AAT​TGC​CAT​TGC​ACA​AC-3′	
*SAA*	5′-TCA​TTT​GTT​CAC​GAG​GCT​TTC-3′	59°C Sultan et al. (2012)
	5′-ATG​GTG​TCC​TCA​TGT​CCT​CTG-3′	
*SNAIL1*	5′-GCG​GAA​GAT​CTT​CAA​CTG​CAA​ATA​TTG​TAA-3′	54°C Li and Siragy (2014)
	5′-GCA​GTG​GGA​GCA​GGA​GAA​TGG​CTT​CTC​AC-3′	
*HPRT1*	5′-TGG​ATA​CAG​GCC​AGA​CTT​TGT​T-3′	59°C Saito et al. (2016)
	5′-CAG​ATT​CAA​CTT​GCG​CTC​ATC-3′	
*TGF-β1*	5′-AAG​GGC​TAC​CAT​GCC​AAC​TT-3′	62°C Wang et al. (2009)
	5′-CGG​GTT​GTG​TTG​GTT​GTA​GA-3′	
*MSR-A2*	5′-AGA​CCT​TCT​GTC​GTT​CCC​CT-3′	57.6°C Pugliese et al. (2001)
	5′-AGA​CCT​TCT​GTC​GTT​CCC​CT-3′	
*Col4A1*	5′-TAG​GTG​TCA​GCA​ATT​AGG​CAG​G-3′	63°C Pugliese et al. (2001)
	5′-TCA​CTT​CAA​GCA​TAG​TGG​TCC​G-3′	
*laminin B1*	5′-CAA​GCT​TGA​GAG​AGG​AAC​GTG​G-3′	52.5°C Pugliese et al. (2001)
	5′-TTA​CCT​TGG​TCA​CCG​AGC-3′	
*RAGE*	5′-AAT​TGT​GGA​TCC​TGC​CTC​TG-3′	59°C Liebisch and Wolf (2020)
	5′-CAG​CTC​TGA​CCG​CAG​TGT​AA-3′	
*TNF*	5′-AAT​TCG​AGT​GAC​AAG​CCT​GTA-3′	60°C Hammerschmidt et al. (2009)
	5′-CTT​GAA​GAG​AAC​CTG​GGA​GT-3′	

NGAL, neutrophil gelatinase-associated lipocalin; KIM1, kidney injury molecule-1; IL-6, interleukin-6; SAA, serum amyloid A; SNAIL1, snail family transcriptional repressor 1; HPRT1, hypoxanthine phosphoribosyl transferase 1; TGF-β1, transforming growth factor-β1; Col4A1, collagen IV, alpha 1; RAGE, receptor for advanced glycation end products, MSR-A2, macrophage scavenger receptor AII; TNF, tumor necrosis factor; Tann, annealing temperature.

### 2.8 Statistical analysis

The data for the male and female mice are presented together to highlight the specificity of the effects and to make basal differences visible. The data are shown in the experiments as the mean ± SD. Scattered dot plots were generated by using GraphPad Prism software (8.4.0). Immune cell profiling data from flow cytometry were analyzed for *p*-value using the non-parametric *t*-test (Mann–Whitney test). In all other data, the significance and interaction were calculated by a three-way ANOVA with genotype (wild-type *vs. RAGE* ^−/−^), sex (male *vs*. female), and age (young *vs*. old) as the three factors. For intergroup significance, Sidak multiple comparisons tests were performed. *p*-values are shown as * above the bar when significant (*p-*value 0.05*, 0.01**, 0.001***, and 0.0001****) and interaction between factors is shown as an inserted graph.

## 3 Results and discussion

### 3.1 Age-dependent increase in AGE receptors and ligands in kidneys

**FIGURE 1:**
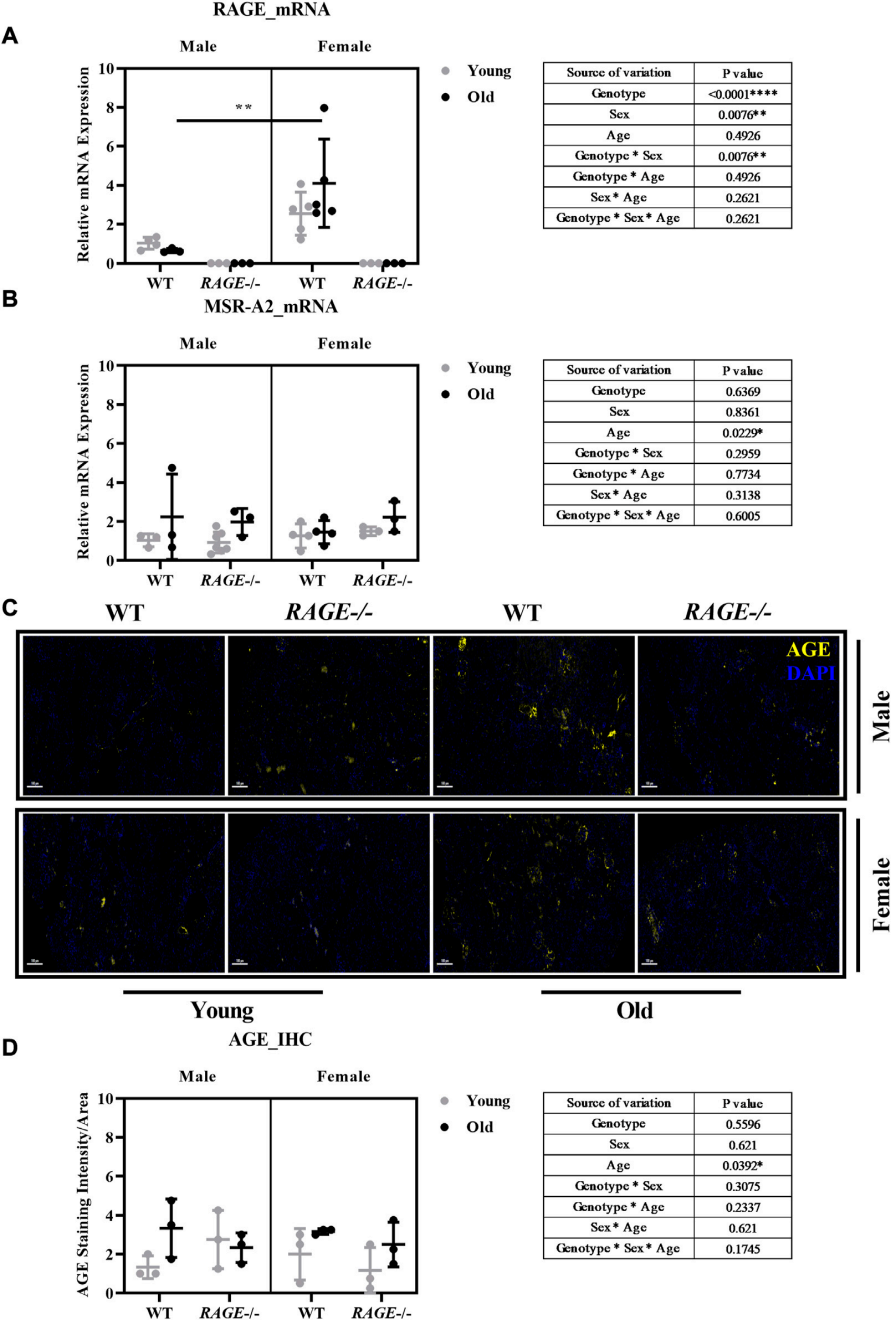
Characterization of AGE receptors and ligands in the kidneys: mRNA quantification by q-PCR for **(A)** RAGE and **(B)** MSR-A2 in the kidney cortex. **(C)** Representative images of AGE staining in the kidney sections in both male and female from the indicated genotypes; yellow indicates protein expressed around the glomerular and tubular region. **(D)** Quantification of AGE staining intensity. Significance and interaction are calculated by three-way ANOVA with genotype (wild type *vs*. RAGE^−/−^, sex (male *vs.* female), and age (young *vs.* old) as three factors (shown in table insert). Sidak multiple comparisons tests were performed for intergroup significance (shown above bars). Young (<30 weeks) and old (>70 weeks) male and female mice (*n* = 3–7) were used in the experiment. Data are shown as mean ± SD; *p-*value: 0.05*, 0.01**, 0.001***, and 0.0001****.

RAGE is a receptor for advanced glycation end products; it binds with several ligands which include AGEs. RAGE after binding to its ligand starts signal transduction pathways and leads to downstream activation of several pro-inflammatory genes. Its role has been implicated in several diseases. RAGE is highly expressed at the development stage in several tissues and decreases over time, but it remains highly expressed in lung tissues (Brett et al., 1993; Katsuoka et al., 1997). Apart from the lungs, its expression is induced in several tissues and cells upon local increase in RAGE ligands (Bierhaus et al., 2005) and pathological inflammatory states (Schmidt et al., 2001). RAGE expression is known to increase in podocytes, tubular/capsular epithelial cells, and vascular smooth muscle cells in the kidneys in human renal biopsies during inflammatory conditions (Bierhaus et al., 1996). We have analyzed RAGE expression at the mRNA level in the kidney cortex of our mouse cohort. There was no product observed in the knockout group with the primer used (Figure 1A). We observed interesting sex-based differences in RAGE expression with old wild-type females showing a significant increase when compared to males, as shown by intergroup comparisons. It is known that estrogen induces the expression of RAGE receptors (Tanaka et al., 2000; Mukherjee et al., 2005), thus this sex-based increase might be due to female hormones, as a protective mechanism for age-induced increase in the AGE content.

In recent years, other receptors of AGEs have been investigated for their possible roles in pathophysiology. MSR is a macrophage scavenger receptor which mediates the endocytic uptake of AGEs *in vivo* in the kidneys (Horiuchi et al., 1996; SMEDSRØD et al., 1997). To study the role of scavenger receptor in aging, we analyzed the mRNA expression of MSR-2A in the renal cortex. Our analysis showed aged-based increase in MSR expression regardless of sex and genotype (Figure 1B), which correlates with age-based increase in AGE content.

Advanced glycation end products (AGEs) and their receptors (RAGEs) not only increase with aging in kidney tissues but also intensify the binding between RAGE and its ligands (Brownlee, 1994; Son et al., 2017). The AGEs-RAGE pathway is a major contributor to kidney aging (Vlassara et al., 2009c). The accumulation of AGEs and decline in renal function during aging may induce the release of inflammatory mediators and the generation of reactive oxygen species (ROS) (Hyogo and Yamagishi, 2008). To investigate AGEs in the kidneys, paraffin sections were stained with AGE antibodies in both males and females in the indicated genotype. Figure 1C shows the representative images of AGE immunofluorescence (stained yellow). AGEs are observed to be accumulated in the glomerular and interstitial regions. There is an age-based significant increase in AGE levels as shown by the three-way analysis of variance (graph insert, Figure 1D). This increase is in accordance with the literature (Son et al., 2017). Although we do not see significant sex differences in AGE accumulation in the *RAGE* wild-type regardless of age, this does not necessarily mean, of course, that blood-soluble RAGE (sRAGE) concentrations are the same for both the sexes. It may also be a result of a complex interplay of sex differences in the circulating sRAGE and AGE clearance in the renal tissue, which should be investigated in more detail in further studies.

### 3.2 Kidney aging phenotype depends on sex and RAGE expression

**FIGURE 2:**
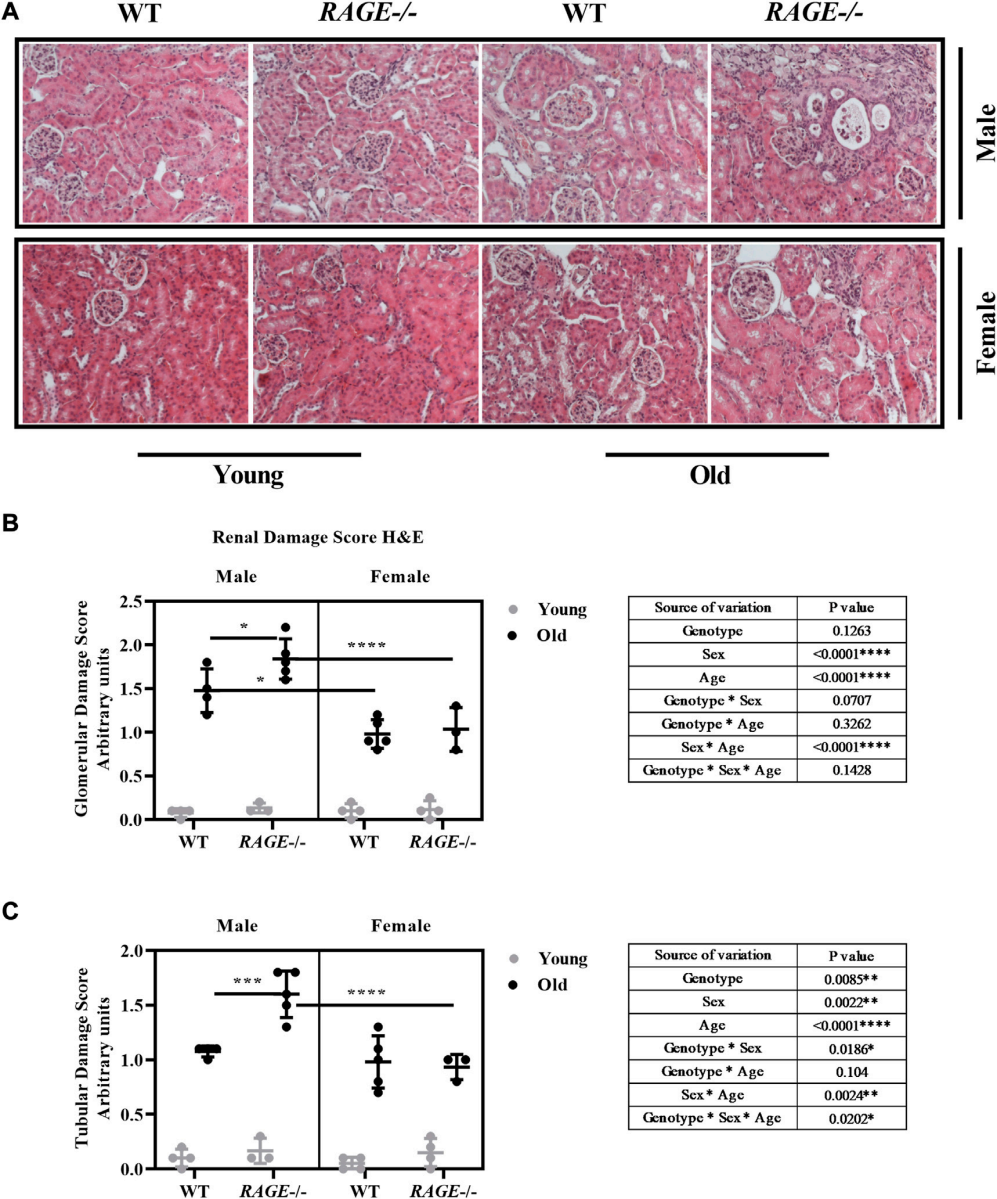
Histopathological characterization for renal damage: **(A)** representative images of kidneys H&E staining from the indicated sex and genotypes, **(B)** quantification of renal glomerular damage, and **(C)** tubular/interstitial damage score. Significance and interaction were calculated by three-way ANOVA with genotype (wild type *vs*. RAGE^−/−^, sex (male *vs.* female), and age (young *vs.* old) as three factors (shown in table insert). Sidak multiple comparisons tests were performed for intergroup significance (shown above bars). Young (<30 weeks) and old (>70 weeks) male and female mice (*n* = 3–5) were used in the experiment. Images were taken under ×200 magnification. Data are shown as mean ± SD; *p-*value: 0.05*, 0.01**, 0.001***, and 0.0001****.

The kidneys were examined histologically using H&E staining, with both glomerular and tubular structural changes as well as the interstitium being analyzed, and Figure 2A shows the representative images of H&E staining in males (upper panel) and females (lower panel) in the indicated genotype and age groups. Another interesting observation is the presence of hyaline casts (data not shown) in the tubules of older KO mice and fewer being observed in old wild-type mice regardless of sex. The presence of casts is generally associated with reduced tubular function and has been implicated in several renal diseases (Ibels and Györy, 1994).

Glomerular (Figure 2B) and tubular damage scores (Figure 2C) show that there is a significant increase in renal damage, which is more significant in males than in females, and this effect is age dependent as well. In addition, a significant interaction was seen between age and sex in both analyses. Intergroup comparison in old wild-type animals has revealed less glomerular damage in female mice, which is consistent with the current findings (Gava et al., 2011). It has been shown that glomerular damage is more severe in aged male than in female C57bl6/J mice (Yabuki et al., 2006). It has been known for 30 years that in experimental models, the male gender enhances the age-related decline in renal function (Bolignano et al., 2014). In a recently published study on sex differences in the loss of kidney function, it was reported that in the general population without major chronic diseases or risk factors for CKD, women exhibited a slower mean GFR decline rate than did men (Melsom et al., 2022). Considering the different genotypes, age-associated renal damage in the glomeruli and tubules increases significantly in male RAGE-KO animals, which is significant when compared with wild-type male mice and also with female RAGE-KO. In the case of tubular analysis, there was a significant interaction between genotype and sex, and also threefold interactions between the factors genotype, sex, and age have been observed.

### 3.3 Deletion of RAGE leads to B-cells infiltration in older kidneys

The immune system functions to maintain renal homeostasis through different effectors which reside in the kidneys. These cells play a vital role in mediating responses to stress or injury. The kidneys consist of unique subsets of immune cells which are distributed in the interstitium and are in cross talk with various other cell types such as the tubular epithelium (Soos et al., 2006; Kaissling and Le Hir, 2008). In normal conditions, the immune cells comprise the macrophages, dendritic cells, neutrophils, and lymphocytes (Nelson et al., 2012; Kolaczkowska and Kubes, 2013; Stewart et al., 2019), however in the diseased compromised state, the composition of the immune cells is altered (Li and Okusa, 2010; Teteris et al., 2011). Stress or damage response chemokines are secreted by injured cells to further attract immune cells from the circulation (Schlöndorff et al., 1997; Chung and Lan, 2011).

**FIGURE 3:**
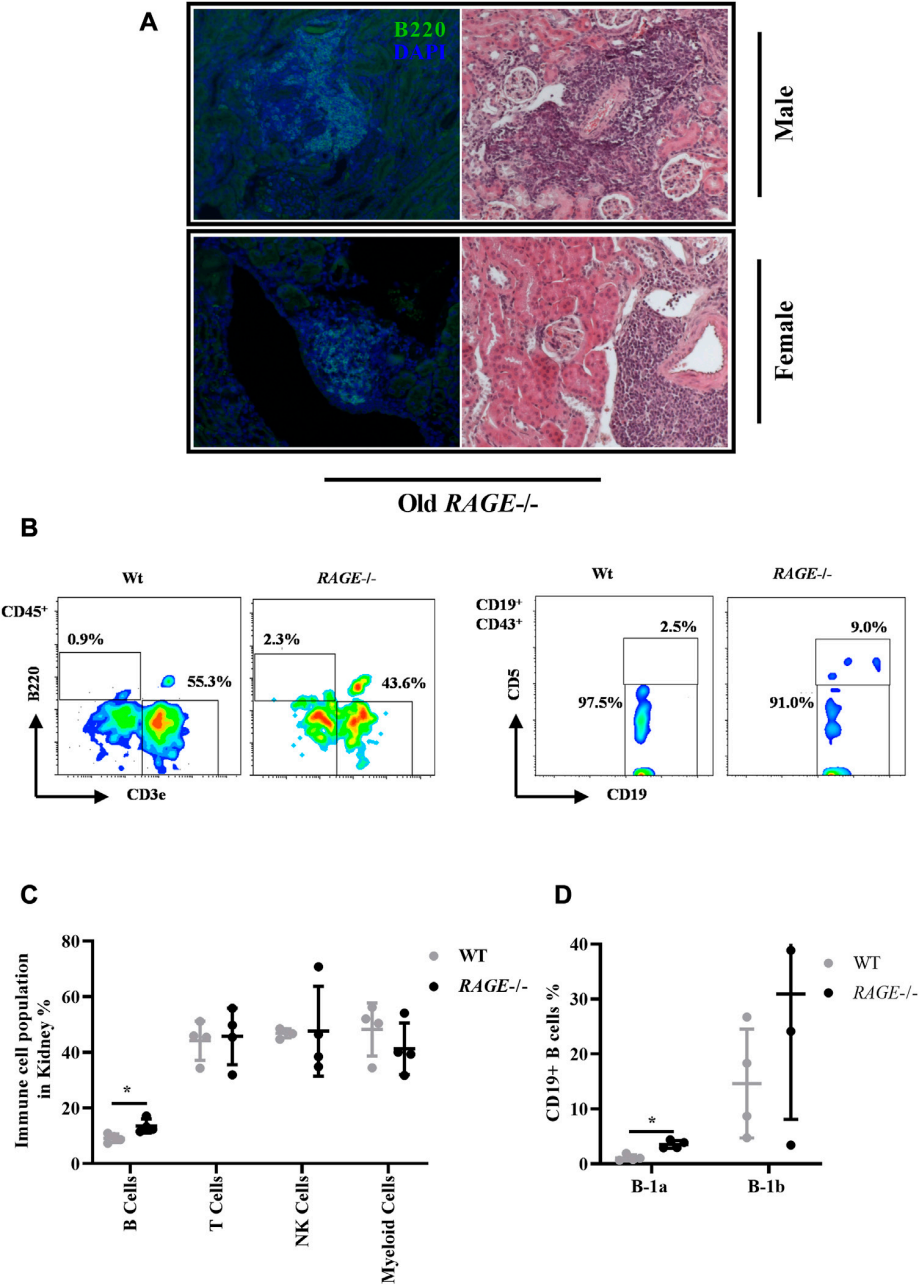
Immune cell profiling of kidney tissues: **(A)** representative images of massive infiltrates in the kidneys from old RAGE^−/−^ male (upper panel) and female (lower panel) mice, and the infiltrates are stained positive for B-cell marker b220 left (green) and right. H&E of the same animal. **(B)** Immune cells were isolated from the whole kidney and analyzed by FACS from 62–68 weeks old wild type and RAGE^−/−^ (*n* = 4) (male and female combined) mice. Representative FACS plots. **(C)** Quantification of B cells (B220), T cells (CD3), NK cells (NK1.1), and myeloid cells (CD11b). Populations were calculated as % of live CD45+ as parent gating. **(D)** B cells were further analyzed for B-1a (CD19+, CD43+, and CD5+) and B-1b (CD19+ and CD43+) subtypes. Population was calculated as % of live C19+ as parent gating. B220, CD19, and B-1a B cells are the dominant population in RAGE^−/−^, and *p*-values were calculated by the non-parametric *t*-test (Mann–Whitney test). Data are shown as mean ± SD; *p-*value: 0.05*, 0.01**, 0.001***, and 0.0001****.

We have observed massive immune infiltrates in both sexes in the old RAGE-KO group (8 out of 10 animals examined have shown infiltration). These infiltrates were mainly located in the extravascular and to fewer accounts, in the extraglomerular space. Immune infiltrates observed in old RAGE-KO in both sexes were stained positive for B220-B cell through immunofluorescence staining (Figure 3A). Older wild-type mice do not show any accumulation of immune infiltrates, indicating that RAGE deletion leads to immune infiltrations in an age-based manner, while this observation is not sex influenced (data not quantified). To better characterize the immune infiltrates observed in older KO animals, we performed a detailed analysis of the immune cells isolated from the kidney tissues through flow cytometric analysis. It is known that immune responses are highly age dependent (Sato and Yanagita, 2019), but studies on the effects of aging on B lymphocytes are conflicting. In most studies, B-cell lymphopenia is a common finding in the healthy elderly (Colonna-Romano et al., 2006; Veneri et al., 2007) but both age-related increase (Colonna-Romano et al., 2006; Morbach et al., 2010) and decrease (Chong et al., 2005; Caraux et al., 2010) in B cells with memory phenotype have been reported.

Figure 3B represents the gating strategy and representative FACS plot. FACS analysis revealed a significant increase in B220-positive B cells in *RAGE*-KO, which is consistent with the staining data (Figure 3C). The mice used in the flow cytometric experiment were 62–68 weeks old, both male and female pooled together due to the limited number of living animals [male (*n* = 3) and female (*n* = 1)], therefore sex-based differences cannot be elucidated due to the small number of animals. The H&E data did not show differences in the occurrences of immune infiltrates between the male and female KO mice. One female mouse in the pooled analysis showed at least no difference when compared to the males. However, we cannot use these findings to argue for sex differences, as there is simply a lack of data; for there might be more infiltration in males and so the renal damage effects could be explained or it could be that infiltrations are the same for both the sexes and the sex differences are to be sought, e.g., in the protective effect of the female sex hormone. Similarly, we do not have younger animals included in the FACS analysis to analyze age-based differences. In addition to B220-positive B cells, a significant increase in the subset of B cell B-1a (CD19+ CD43+ CD5+) (Figures 3C, D) has also been observed in RAGE-KO mice when compared to the wild type. B lymphocytes are subdivided into B-1a and B-1b cells. B-1a lymphocytes represent the largest B-cell population in early life (Kantor, 1991) and produce most of the natural IgM antibodies in the serum before the development of B-2 cells. B-1b cells are complementary to B-1a lymphocytes in that they protect the body from parasites and bacteria, a function minimally provided by B-1a cells (Alugupalli and Gerstein, 2005; Haas et al., 2005). A decrease has been reported with age (Hannet et al., 1992), while increased levels have been found in autoimmune diseases (Höffkes et al., 1996; Weksler and Szabo, 2000; Dono et al., 2004). Consistent with the literature, an increase was observed in B-1a cells in KO mice underlying existing renal pathology and altered immune responses.

Other immune cells have also been analyzed such as CD3^+^ T cells, NK cells, and myeloid cells and shown no significant difference (Figure 3C). Dendritic cells, eosinophils, basophils, neutrophils, and kidney macrophages were also analyzed and have shown no significant differences between the groups (data not shown).

### 3.4 Age-induced expression of pro-fibrotic and pro-inflammatory markers differs between sex and RAGE genotypes

**FIGURE 4:**
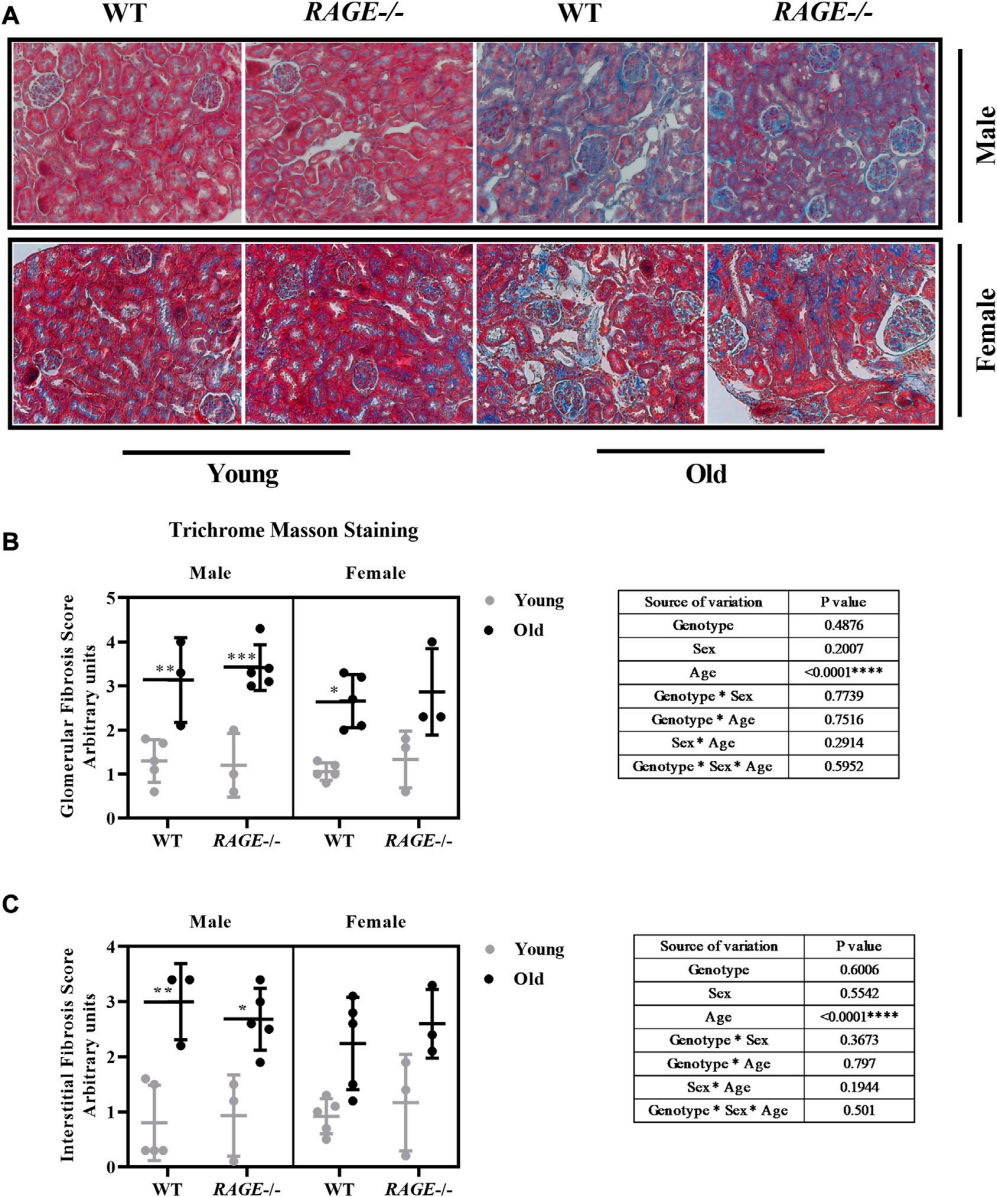
Renal fibrosis score assessment through Masson’s trichome staining: **(A)** representative images of Masson’s trichome staining of kidney sections from the indicated genotypes. Blue area indicates elevated fibrosis, **(B)** quantification of renal glomerular fibrosis, and **(C)** tubular/interstitial fibrosis scores. Significance and interaction was calculated by three-way ANOVA with genotype (wild type *vs.* RAGE^−/−^, sex (male *vs.* female), and age (young *vs.* old) as three factors (shown as table insert). Sidak multiple comparisons tests were performed for intergroup significance (shown above bars). Young (<30 weeks) and old (>70 weeks) male and female mice (*n* = 3–7) were used in the experiment. Images were taken under ×200 magnification. Data are shown as mean ± SD; *p-*value: 0.05*, 0.01**, 0.001***, and 0.0001****.

To investigate the accumulation of the extracellular matrix (ECM), following immune infiltration, the fibrosis score was calculated using Trichrome Masson staining and representative images (male upper panel, female lower panel) are shown (Figure 4). Visual quantification of the blue collagen and ECM was done in both the glomeruli (Figure 4B) and interstitium (Figure 4C). Both wild-type and KO mice showed highly significant increases in fibrosis score in an age-dependent manner, which is in line with the published data from several groups. It is well known that immune cell infiltration leads to inflammation and subsequent accumulation of the extracellular matrix (ECM) in the tissues. This contributes to the senescence phenotype, which is one of the hallmarks of aging, and the process is known as inflammaging (Collado et al., 2007; Franceschi and Campisi, 2014; Sepe et al., 2022). Except for a highly significant age effect, we did not detect differences in the collagen content between the two sexes or genotypes in either the glomeruli or interstitium. The examined cohort might be small, and/or Masson staining alone is not sensitive enough to elucidate further differences.

**FIGURE 5:**
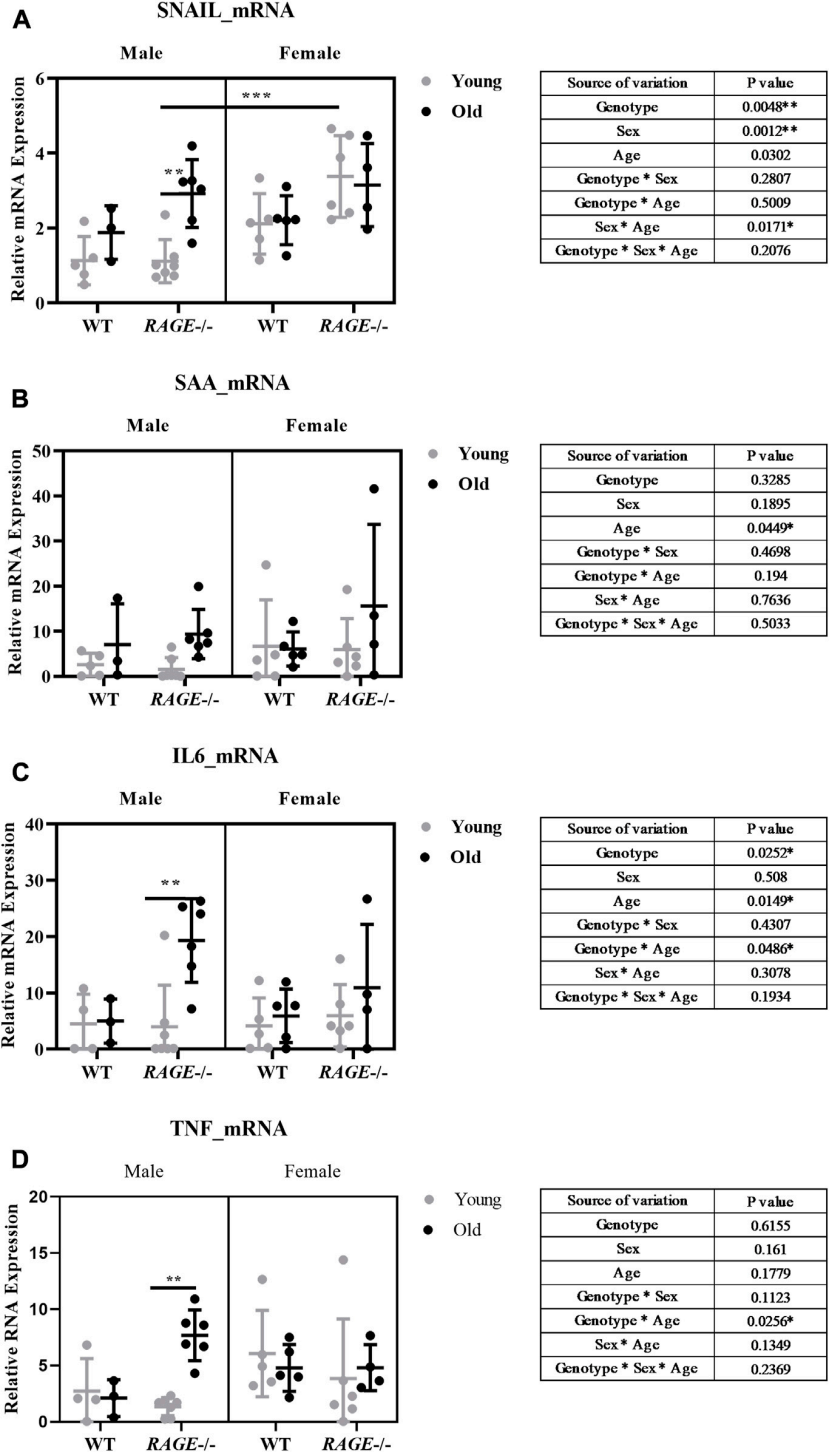
Expression of pro-fibrotic/pro-inflammatory markers in the mouse kidney: mRNA quantification by q-PCR for **(A)** SNAIL, **(B)** SAA, **(C)** IL-6, and **(D)** TNF. Significance and interaction were calculated by three-way ANOVA with genotype (wild type *vs.* RAGE^−/−^, sex (male *vs.* female), and age (young *vs.* old) as three factors (shown as table insert). Sidak multiple comparisons tests were performed for intergroup significance (shown above bars). Young (<30 weeks) and old (>70 weeks) male and female mice (*n* = 3–7) were used in the experiment. Data are shown as mean ± SD; *p-*value: 0.05*, 0.01**, 0.001***, and 0.0001****.

**FIGURE 6:**
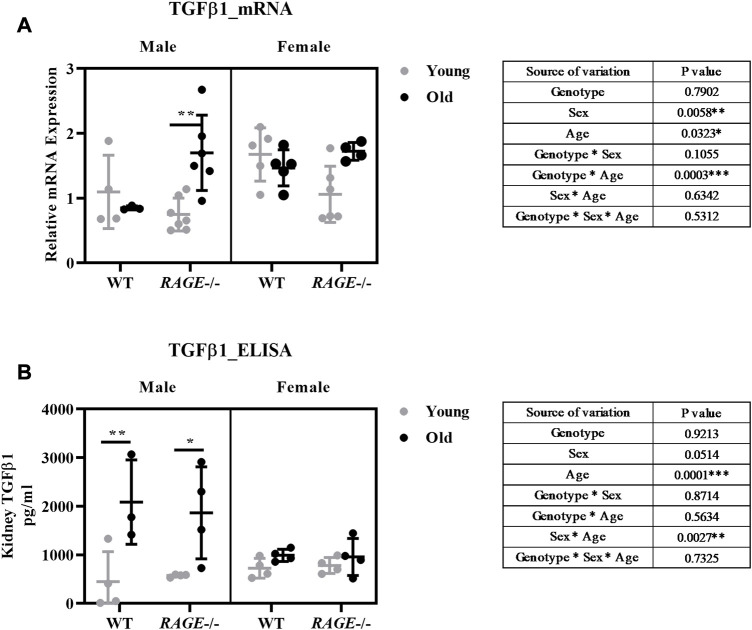
Expression analysis of TGF-β1 at the mRNA and protein level: **(A)** mRNA quantification by q-PCR for TGF-β1 markers from the renal cortex. **(B)** Quantification of TGF-β1 protein concentrations in kidney tissue homogenates by ELISA. Significance and interaction were calculated by three-way ANOVA with genotype (wild type *vs.* RAGE^−/−^, sex (male *vs.* female), and age (young *vs.* old) as three factors (shown in table insert). Sidak multiple comparisons tests were performed for intergroup significance (shown above bars). Young (<30 weeks) and old (>70 weeks) male and female mice (*n* = 3–7) were used in the experiment. Data are shown as mean ± SD; *p-*value: 0.05*, 0.01**, 0.001***, and 0.0001****.

Thus, we examined kidney fibrosis in more detail on the basis of mRNA expression patterns of pro-inflammatory and pro-fibrotic markers (Figures 5, 6). Snail, a transcription factor important for pro-inflammatory/pro-fibrotic gene expression, showed enhanced expression in old KO male mice when compared to wild-type and young KO mice. Females, on the other hand, did not show differences between the young and old KO groups but interestingly showed more overall expression than their male counterpart (Figure 5A). The renal mRNA expression of the acute phase protein SAA (serum amyloid A), which is, in addition to its dissemination by systemic circulation, also produced locally at inflammation sites (Anderberg et al., 2015), is upregulated in the old groups, independent of sex and genotype (Figure 5B). Other pro-inflammatory markers IL-6 (Figure 5C), TNF (Figure 5D), and TGF-β1 (Figure 6A) showed significant expressions in old male KO mice when compared with the rest of the groups. Also, the analysis of variance revealed several factor interactions: genotype*age interaction for IL6 (Figure 5C), TNF (Figure 5D), and TGF-β1 (Figure 6A) and sex*age interaction for SNAIL1 (Figure 5A). There is a mixed expression of pro-fibrotic and pro-inflammatory factors, except for the expression of SAA (Figure 5B); it is always the combination of male sex and the knockout of RAGE that leads to significant upregulation of markers in older animals (see intergroup comparisons male-KO-young *vs*. male-KO-old). It is known that the age-induced increase in inflammatory factors, especially IL-6, TNF, and IL-1, is believed to accelerate renal tissue destruction (Vasto et al., 2007; Baylis et al., 2013) and possibly explain the overall renal pathology in old male KO mice. This expression gives us a brief overview and to further validate the result, protein expression might be important. In the case of TGF-β1, we performed renal protein lysate analysis and found a significant age effect in males and a sex*age interaction (Figure 6B). Contrary to the mRNA analysis, there was no difference between old wild-type and knockout males. However, it should be noted that at the protein level, a distinction is made between latent/inactive and biologically active TGF-β1, but the ELISA test performed could not distinguish between the two forms.

### 3.5 RAGE and sex influence age-driven renal fibrosis

**FIGURE 7:**
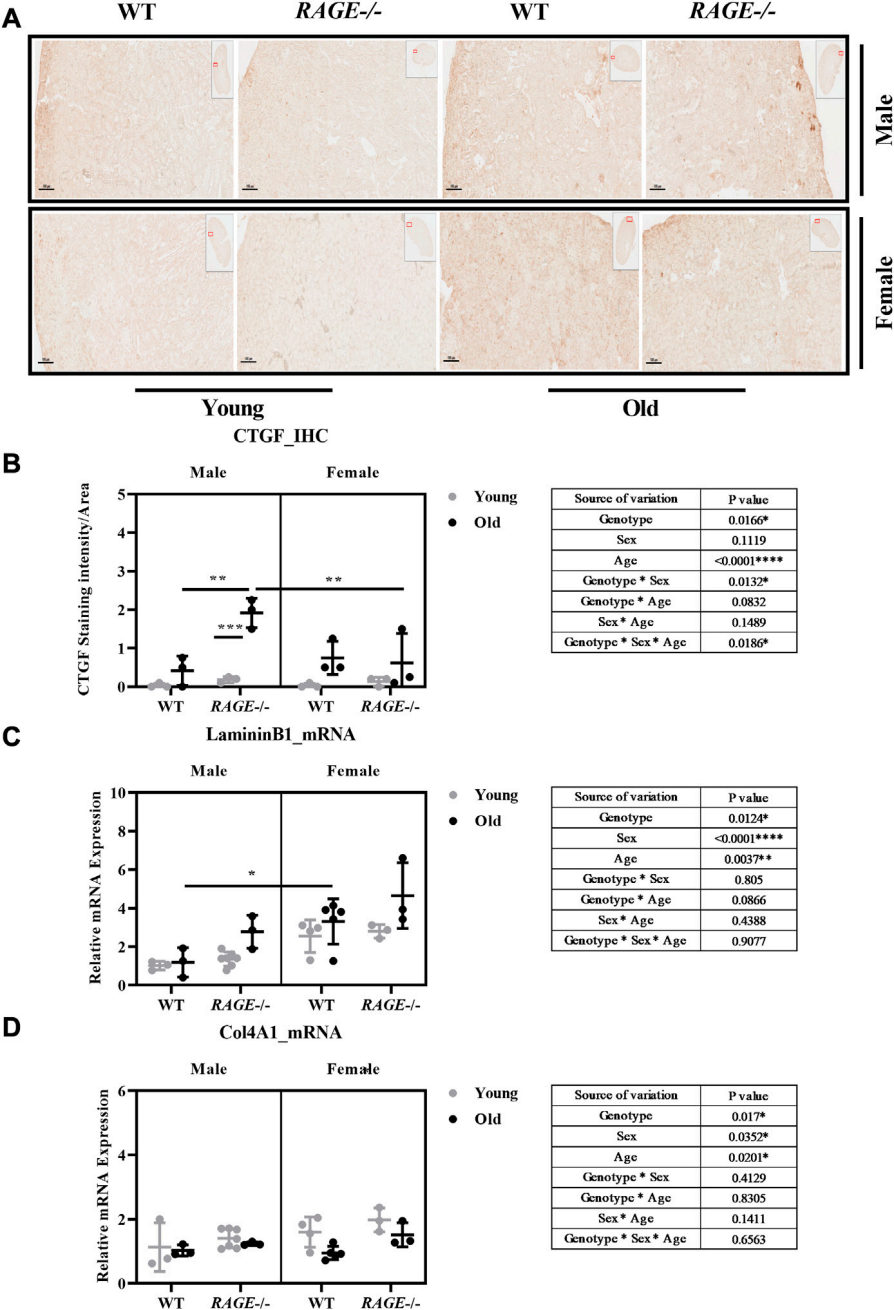
Quantification of kidney fibrosis: **(A)** representative images of CTGF immunohistochemistry in kidney sections from the indicated genotypes. Brown area indicates CTGF staining and **(B)** quantification of CTGF staining intensity. mRNA quantification by q-PCR for **(C)** *laminin B1* and **(D)** *Col4A1*. Significance and interaction were calculated by three-way ANOVA with genotype (wild type *vs* RAGE^−/−^, sex (male *vs.* female), and age (young *vs.* old) as three factors (shown in table insert). Sidak multiple comparisons tests were performed for intergroup significance (shown above bars). Young (<30 weeks) and old (>70 weeks) male and female mice (*n* = 3–7) were used in the experiment. Images were taken under ×100 magnification. Data are shown as mean ± SD; *p-*value*:* 0.05*, 0.01**, 0.001***, and 0.0001****.

To better characterize fibrosis, we performed staining for connective tissue growth factor (CTGF) and analyzed mRNA expression of laminin B1 and collagen IV alpha 1 (Col4A1) (Figure 7). We assessed age-induced fibrosis (CTGF) as weak to mild on the basis of this staining. Again, the strongest induction was seen in male *RAGE* knockout mice as was in the sex*genotype interaction (Figure 7A).

Interestingly, the mRNA analyses of laminin B1 and Col4A1 do not display entirely the expected pattern of expression, which should actually be consistent with the pattern of the pro-fibrotic factors SNAIL1 and TGF-β1. For laminin B1, the data are as expected in the male sex but differ significantly in the female sex (Figure 7C). It is striking that laminin B1 is more highly expressed in all female groups (Figure 7C) and collagen IV in young females(Figure 7D) than in males. A very recent study showed sexual dimorphism in the ECM composition of the healthy mouse brain and demonstrated a significantly higher mRNA expression of laminin and collagen type IV in the healthy female cerebral cortex (Batzdorf et al., 2022). Whether the expression shown here is due to SNAIL1 and TGF-β1–independent mechanisms should be analyzed in more detail in future studies. Furthermore, to obtain a complete picture, all matrix components contributing to fibrosis in the tissues would have to be investigated.

### 3.6 RAGE and sex influence age-driven upregulation of kidney damage markers

**FIGURE 8:**
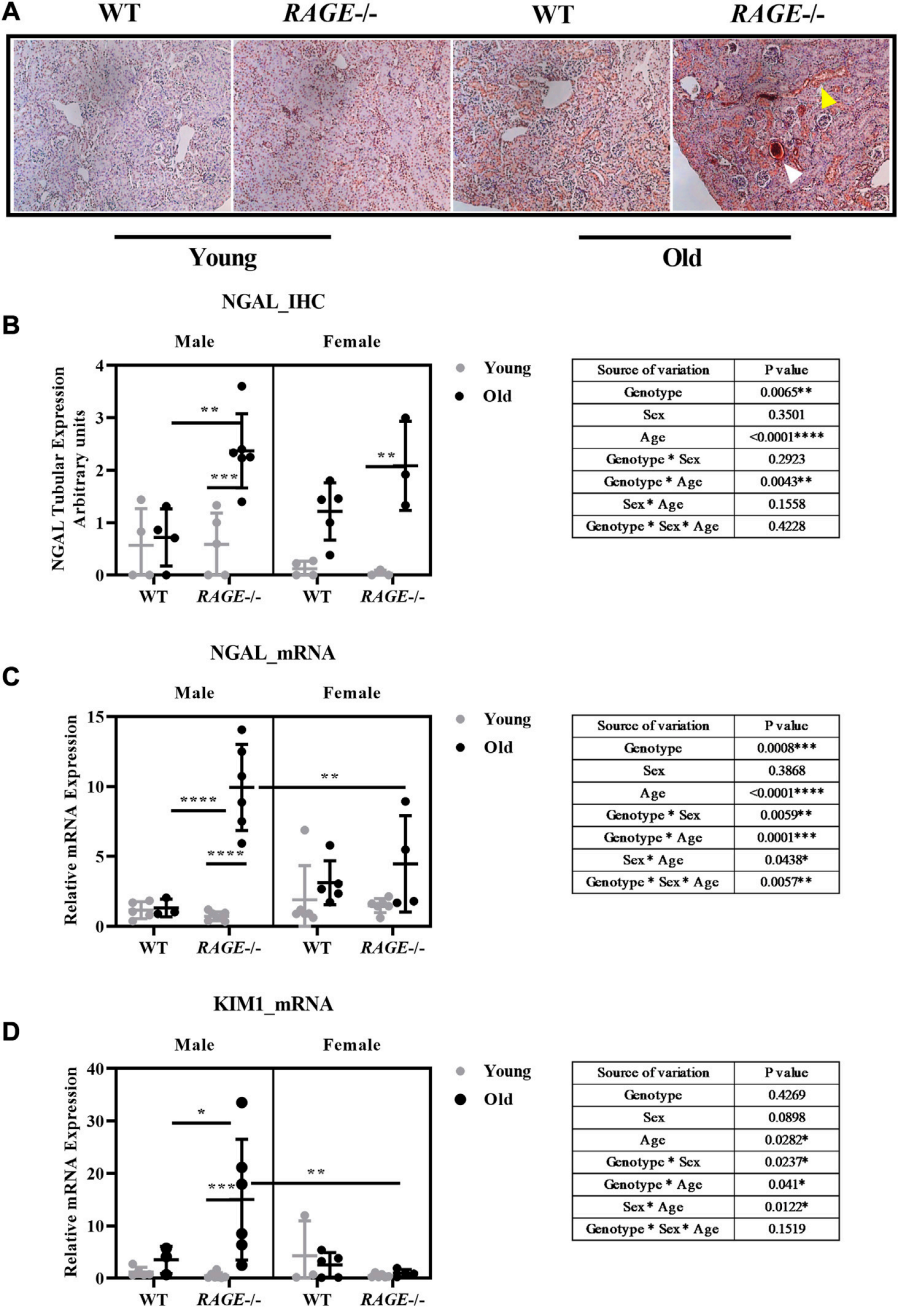
Renal damage marker expression at the RNA and protein level: **(A)** representative images of NGAL immunohistochemistry from the indicated genotypes. Yellow arrows indicate reddish brown NGAL tubular staining, and white arrows indicate intensely stained protein cast in old KO. **(B)** Quantification of NGAL IHC, **(C)** mRNA expression by q-PCR for NGAL, and **(D)** KIM1 damage markers. Significance and interaction were calculated by three-way ANOVA with genotype (wild type *vs.* RAGE^−/−^, sex (male *vs.* female), and age (young *vs.* old) as three factors (shown in table insert). Sidak multiple comparisons tests were performed for intergroup significance (shown above bars). Young (<30 weeks) and old (>70 weeks) male and female mice (*n* = 3–7) were used in the experiment. Images were taken under ×200 magnification. Data are shown as mean ± SD; *p-*value*:* 0.05*, 0.01**, 0.001***, and 0.0001****.

In addition to immune cell infiltrations in the kidneys from old RAGE-KO mice, H&E staining also revealed sex differences in glomerular and tubular damage (Figures 2B, C). To further investigate this, we tested the effects of sex and RAGE genotype on specific markers for kidney damage, in particular neutrophil gelatinase-associated lipocalin (NGAL/Lcn2) and kidney injury molecule-1 (KIM1) (Figure 8). NGAL is a lipocalin iron-carrying protein of 25 kDa and is expressed by renal tubular epithelial cells following tubulointerstitial injury (Lopez-Giacoman and Madero, 2015). Immunohistochemistry for NGAL staining (Figure 8A) showed the strongest expression in renal tubules which were stained dark brown in old RAGE-KO animals. Multifactorial analysis of variance revealed a significant interaction between genotype and age for NGAL expression at the protein (Figure 8B) and mRNA (Figure 8C) levels. An automated quantification of immunohistochemistry yielded the same results as the visual analysis with an additional genotype*sex interaction (Supplementary Figure S1). While sex differences were difficult to detect at the protein level, the qPCR data showed significantly less NGAL mRNA in the kidneys from old female RAGE-KO animals when compared with old male RAGE-KO animals (see graph intergroup comparison in Figure 8C). This is reflected in a significant threefold interaction between the factors genotype, age, and sex (see table inserts in Figure 8C).

The expression analysis of KIM1 mRNA (Figure 8D) revealed a similar picture as was for NGAL mRNA: a strong age-dependent induction in male RAGE-KO animals; however, in female animals, regardless of the genotype, and in male wild type, the expression was negligible. Overall, the expression changes of NGAL and KIM1 in our old wild-type mice are not very strong, which seems contradictory to the data from the study using similarly aged mice (Braun et al., 2016). The increase in these renal damage markers at the transcriptomic and protein levels is moderate but significant. A very likely explanation of these differences between the two studies may be the genetic backgrounds of the animals used (mixed C57BL/6; 129S5/SvEvBrd; C57BLKS in our study and mixed FVB/C57BL6 in their study), which may influence susceptibility to age-induced renal injury.

Various studies have addressed this problem of genetic background (Ma and Fogo, 2003; Schmitt et al., 2009; Lu et al., 2012; Bufi and Korstanje, 2022). It has been demonstrated that genetic background has a major impact on the phenotype of kidney disease. Different mouse strains and sub-strains exhibit a range of severities in kidney disease (Bufi and Korstanje, 2022), whereas C57BL/6 strains are relatively resistant to kidney damage; 129/Sv is the background with robust induction of glomerulosclerosis and tubulointerstitial fibrosis in the remnant kidney model (Ma and Fogo, 2003). In contrast to this, however, is another study that has shown that C57BL/6 mice are much more sensitive to damage caused by renal ischemia–reperfusion than are 129/Sv mice (Lu et al., 2012). Interestingly, also differences between the C57BL/6 sub-strains have implications in kidney disease phenotypes (Bufi and Korstanje, 2022). For example, in aging mice, Schmitt et al. (2009) evaluated the baseline renal parameters and morphology of 19- to 22-month-old B6J mice purchased from four different suppliers; while normal renal aging was present in mice from three providers, they found unexpectedly high serum urea and proteinuria in mice from one supplier, which was associated with massive glomerulosclerosis and increased tubulointerstitial fibrosis in the kidneys (Schmitt et al., 2009). Of high importance to our study is a recently published study that used the same strain (B6;129S5-Ager<tm1Lex>/leg) and examined a similar age (Teissier et al., 2019). Therefore, RAGE-knockout mice that were protected against nephrosclerosis exhibited less inflammation and were better protected against oxidative stress (Teissier et al., 2019), which is the opposite of our findings. We can explain this by the different genetic backgrounds: while we worked with littermates with mixed background, it is clear from the methodological descriptions of Teissier et al. (2019) that they obtained RAGE-knockout animals from a group at the New York University and compared them with WT controls from Janvier Labs and not with littermates.

## 4 Conclusion

Taken together, the aim of this study was to investigate potential RAGE-dependent sex differences in aging kidneys. Interestingly, the male knockout animals exhibited more severe renal damage and fibrosis than the animals expressing RAGE. We can say that in our animals, male sex together with RAGE-KO accelerates the occurrence of renal damage typical of aging (premature aging). We rather expected a reno-protective effect of knockout, since RAGE expression has been shown to be associated with renal fibrosis (Gasparitsch et al., 2013; Sanajou et al., 2018). It has been shown that accumulation of RAGE triggers oxidative stress and inflammation, which is the major deleterious effect of AGEs in the host and intestinal microenvironment of aging conditions; therefore, targeting RAGE is considered as an additional intervention strategy for aging kidneys (Wu et al., 2021). AGEs are cleared from the body in the receptor and non-receptor–mediated pathways by the cellular proteolytic system (Vlassara, 2001). At the tissue level, liver (SMEDSRØD et al., 1997; Nagai et al., 2007) and kidneys (Miyata et al., 1998) are involved in the clearance mechanism. Kidney function has been implicated in AGEs clearance and thus as an important site for AGE-induced pathology (Li et al., 1996b; Forbes et al., 2003; Tan et al., 2007; Harcourt et al., 2011).

We explain the opposite results by the high variability of genetic backgrounds and control animals used in the literature. However, since we studied littermates for both the wild-type control groups and different sexes, the differences in genetic backgrounds neutralize, and equally important, the environment is also comparable (Holmdahl and Malissen, 2012). Moreover, accumulated AGEs in older animals may bind to other receptors in the absence of RAGE initiating proinflammation and renal damage. Also, AGEs, in the absence of RAGE, may directly bind to cellular structures such as histones and play an important role in pathogenesis of inflammatory responses (Itakura et al., 2022).

Apart from the membranous form on various cell surfaces, RAGE is also present in a soluble splice form (sRAGE) (Schmidt et al., 2000; Hudson et al., 2008). This secreted isoform is described as a potential competitor of membrane-bound RAGE and its ligand interaction, possibly blocking the deleterious effects of the interaction (Lee and Park, 2013). This might explain the possible protection in old wild-type animals where sRAGE binds to circulating age-induced AGEs, thus preventing it from downstream activation of signaling cascades. However, this protective mechanism is absent in old KO mice in our study, leading to accelerated renal damage. There are other receptors to which circulation AGEs can bind, thus preventing additional damage to the RAGE–AGE interaction. One of this receptor is AGER1, also known as OCT48, which lowers AGEs and facilitates its clearance into urine (Vlassara et al., 2009b; Uribarri et al., 2011) and is known as an anti-inflammatory AGE receptor (Lu et al., 2004); it is downregulated in many autoimmune diseases such as CDK and diabetes (Stinghen et al., 2016). Although AGER1 receptor’s over-expression clears AGE burden, it leads to tubulointerstitial damage and fibrosis (Zhuang et al., 2019). Similarly, when overexpressed in mice podocytes, glomerulosclerosis and podocyte injury result despite AGE clearance (Zhuang et al., 2021). Similarly, the role of the macrophage scavenger receptor (MSR) has been implicated in the uptake and removal of AGEs from the circulation in the kidneys. We can say that different backgrounds and other possible receptor interactions with AGEs might explain the differences with published studies.

Despite all the accumulated data, the underlying mechanism of accumulated damage in our mouse model is unclear, and further detailed studies have to be elucidated with advanced techniques, bigger animal cohort, and different age groups. Furthermore, complex interactions with other receptors might give interesting insights.

## Data Availability

The raw data supporting the conclusion of this article will be made available by the authors, without undue reservation.

## Supplementary material

SUPPLEMENTARY FIGURE S1 NGAL immunohistochemistry quantification by an automated method from the indicated genotypes. Significance and interaction was calculated by three-way ANOVA with genotype (wild type *vs*. RAGE^−/−^, sex (male *vs*. female), and age (young *vs*. old) as three factors (shown as table insert). Sidak multiple comparison tests were performed for intergroup significance (shown above bars). Young (<30 weeks) and old (>70 weeks) male and female mice (*n* = 3–4) were used in the experiment. Images were taken under ×200 magnification. Data are shown as mean ± SD; *p*-value 0.05*, 0.01**, 0.001***, and 0.0001****.

## Abbreviation

AGEs, advance glycation end products; AKI, acute kidney damage; BSA, bovine serum albumin; CD, cluster of differentiation; CKD, chronic kidney disease; Col4A1, collagen IV DAPI (4′,6-diamidino-2-phenylindole); ECM, extracellular matrix; ELISA, enzyme-linked immunosorbent assay; GFR, glomerular filtration rate; HbA1c, glycated hemoglobin; H&E, hematoxylin and eosin; HPRT1, hypoxanthine phosphoribosyltransferase 1; IL-6, interleukin-6; KIM1, kidney injury molecule-1; KO, knockout; MSR-2A, macrophage scavenger receptor 2A; NGAL, neutrophil gelatinase-associated lipocalin; NIH, National Institute of Health; NK1.1, natural killer cells; PBS, phosphate-buffered saline; PCR, polymerase chain reaction; RAGE, receptor for advanced glycation end product; ROS, reactive oxygen species; SAA, serum amyloid A; SD, standard deviation; SNAIL1, snail family transcriptional repressor 1; Tann, annealing temperature; TNF, tumor necrosis factor; TGF-β1, transforming growth factor β.
