# Supplementary figures and images for: RAGE is a critical factor of sex-based differences in age-induced kidney damage
Source: Front Physiol. 2023 Mar 29;14:1154551. doi: 10.3389/fphys.2023.1154551 (PMC10090518; doi:10.3389/fphys.2023.1154551)

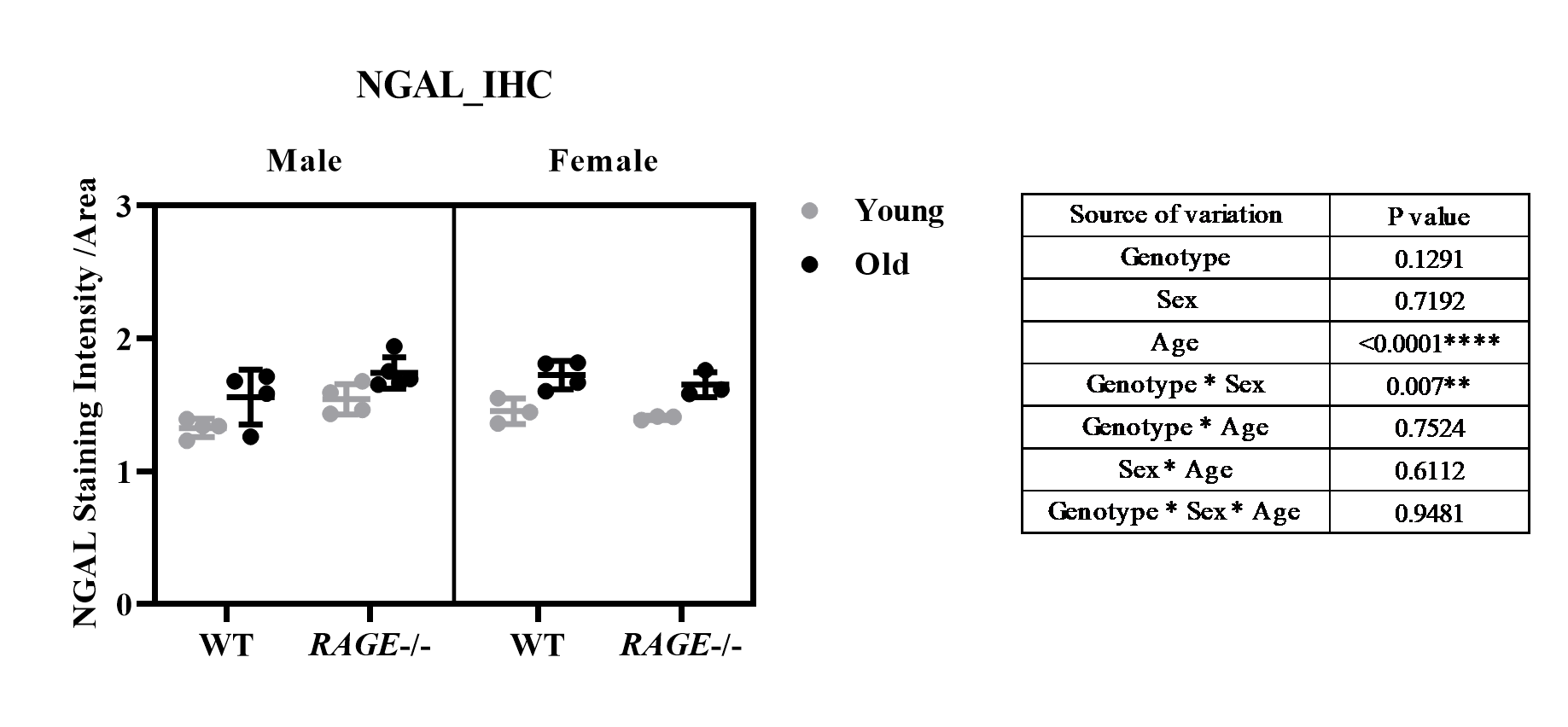

Supplement: Supplementary file 1 [file Image1.TIF]
